# Correction to ‘DNA extrusion size determines pathway choice during CAG repeat expansion’

**DOI:** 10.1093/nar/gkag061

**Published:** 2026-01-22

This is a correction to: Mayuri Bhatia, Ashutosh S Phadte, Anna Lakhina, Anthony R Monte Carlo III, Sarah Barndt, Anna Pluciennik, DNA extrusion size determines pathway choice during CAG repeat expansion, *Nucleic Acids Research*, Volume 53, Issue 22, 11 December 2025, gkaf1393, https://doi.org/10.1093/nar/gkaf1393

In the originally published version of this article, four supplementary tables (Supplementary Tables 1–4) were inadvertently omitted from the online materials. These tables have now been added to the Supplementary Data section.

The publisher apologizes for the oversight. This correction does not affect the findings or conclusions of the article.

