# Supplementary material for: DNA extrusion size determines pathway choice during CAG repeat expansion
Source: Nucleic Acids Res. 2025 Dec 22;53(22):gkaf1393. doi: 10.1093/nar/gkaf1393 (PMC12721328; doi:10.1093/nar/gkaf1393)
Supplement: gkaf1393_Supplemental_Files [file gkaf1393_supplemental_files.zip › Bhatia et al. Supplementary Information.pdf]

DNA extrusion size determines pathway choice during CAG repeat expansion

Mayuri Bhatia[1], Ashutosh S. Phadte[1], Anna Lakhina[1], Anthony R. Monte Carlo III[1], Sarah Barndt[1] and Anna Pluciennik[1,*]

[1] Department of Biochemistry and Molecular Biology, Sidney Kimmel Medical College, Thomas Jefferson University, Philadelphia, PA, 19107, USA

SUPLEMENTARY MATERIALS

Supplementary Figure 1-5

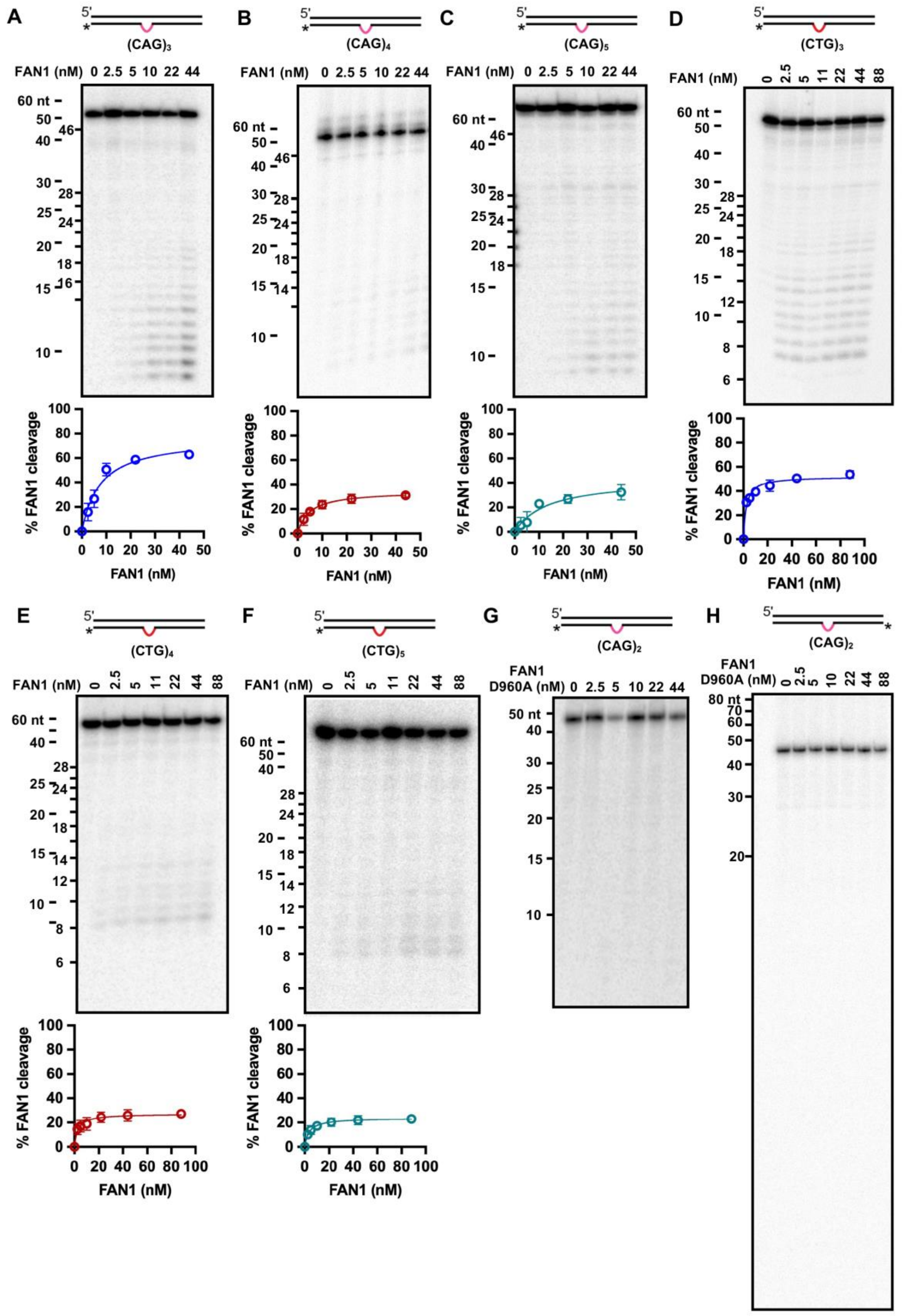

Supplementary Figure 1. FAN1 cleavage activity is governed by the size of the extrahelical extrusion. 3'-end radiolabeled DNA substrates harboring (**A**) $(CAG)_3$, (**B**) $(CAG)_4$, (**C**) $(CAG)_5$, (**D**) $(CTG)_3$, (**E**) $(CTG)_4$, (**F**) $(CTG)_5$ were incubated with increasing concentrations of FAN1 at 37ºC in presence of 70 mM KCl and 5 mM $MgCl_2$ for 10 min (see Materials and Methods). Samples were collected at indicated

time points and resolved on 20% denaturing PAGE. Representative images are shown. Quantification of the percentage of FAN1 cleavage is shown below each respective image. (**G, H**) Five nM 3'-end radiolabeled or 5'-end radiolabeled $(CAG)_2$ DNA substrate, respectively, were incubated with increasing concentrations of a catalytically dead mutant of FAN1 (D960A) at 37ºC in presence of 70 mM KCl and 5 mM $MgCl_2$ for 10 min. Samples were collected analyzed as above. Graphs are presented as mean values ± SD of at least 3 independent experiments.

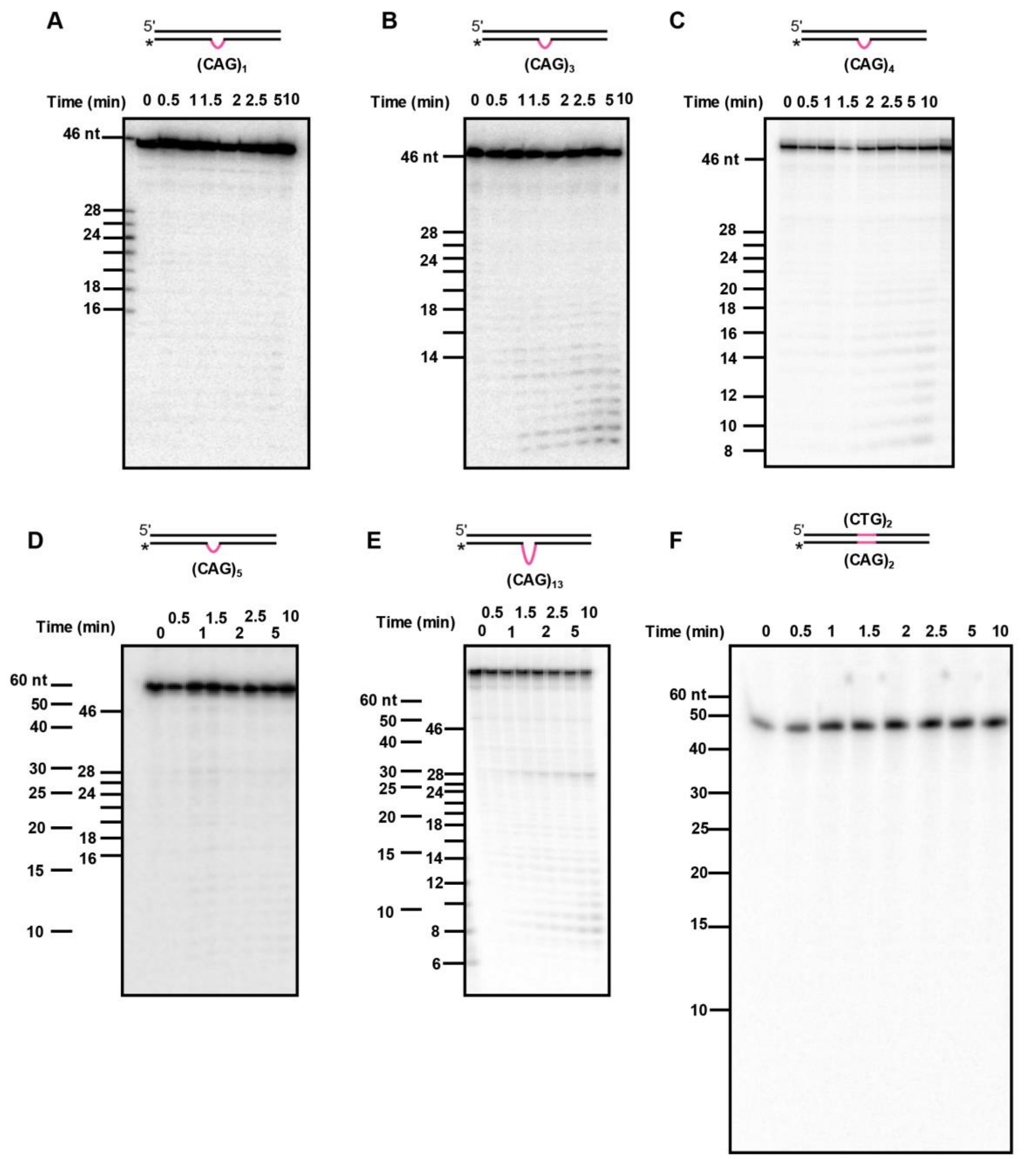

Supplementary Figure 2. FAN1 time dependent catalysis on the CAG extrusions. Five nM of 3'-radiolabeled DNA substrates harboring (**A**) $(CAG)_1$, (**B**) $(CAG)_3$, (**C**) $(CAG)_4$, (**D**) $(CAG)_5$, (**E**) $(CAG)_{13}$, or (**F**) homoduplex control were incubated with 11 nM FAN1 at 37ºC in presence of 70 mM KCl and 5 mM $MgCl_2$. Samples were collected at indicated time points and resolved on 20% denaturing PAGE. The images are representatives of n=3 independent experiments (with error bars representing SD).

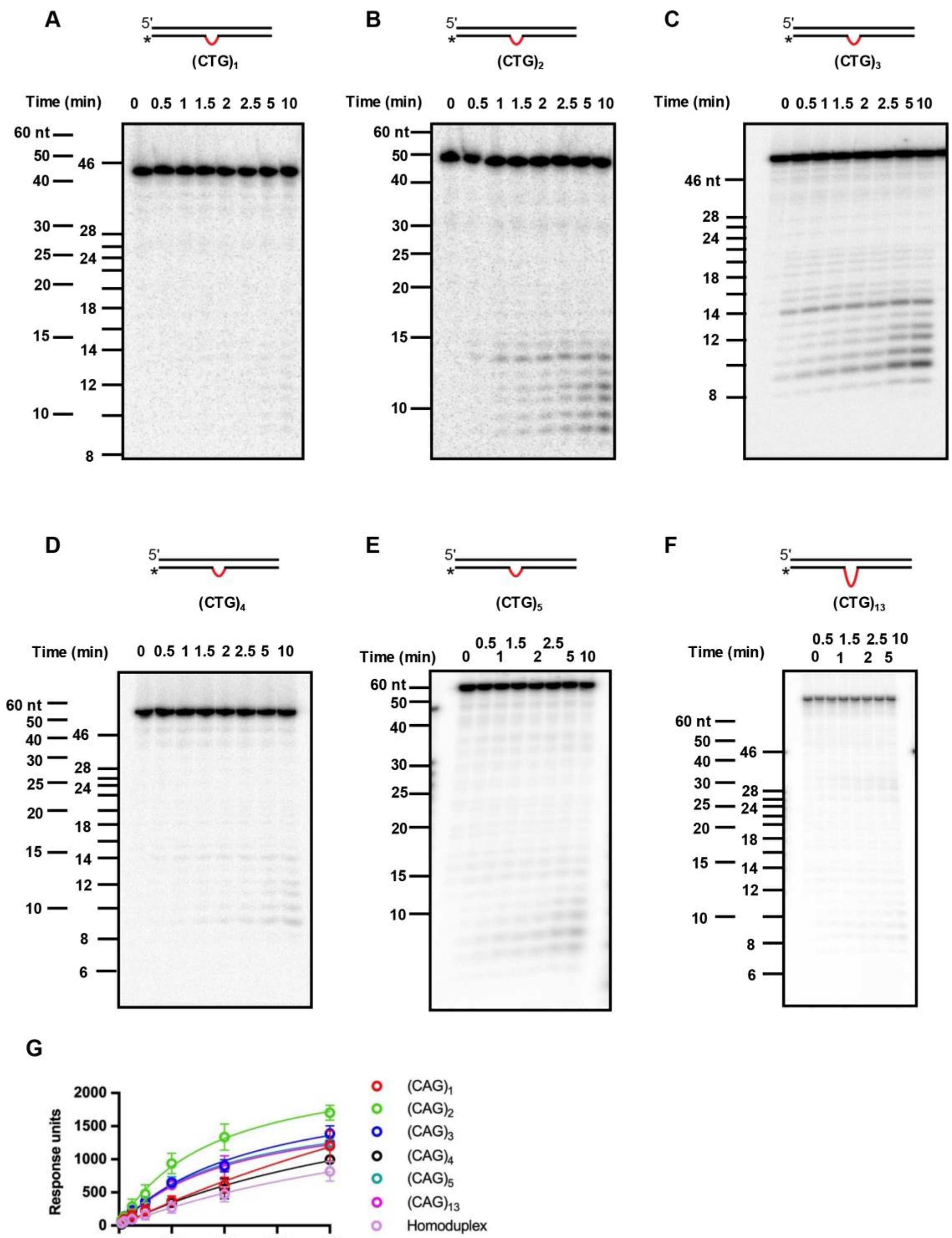

Supplementary Figure 3. FAN1 time dependent catalysis on the CTG extrusions. Five nM of 3'-radiolabeled DNA substrates harboring (**A**) $(CTG)_1$, (**B**) $(CTG)_2$, (**C**) $(CTG)_3$, (**D**) $(CTG)_4$, (**E**) $(CTG)_5$, or (**F**) $(CTG)_{13}$ were incubated with 11 nM FAN1 at 37ºC in the presence of 70 mM KCl and 5 mM $MgCl_2$. Samples were collected at indicated time points and resolved on 20% denaturing PAGE. The images are representative of n=3 independent experiments. (**G**) The assembly of FAN1-DNA binary complex was scored by SPRS using a sensor chip derivatized with 40-bp DNA substrates harboring $(CAG)_{1,2,3,4,5,13}$ extrusion. Apparent affinity of FAN1 for different DNA substrates was determined from SPRS experiments like those described in (Figure 2E, F). The data were fit to a hyperbola using Eq (1) (Materials and Methods). We observed a substantial FAN1 binding to homoduplex control, although the

binding curves do not saturate. Data are based on n≥3 independent experiments with error bars representing SD. Data were also plotted after subtraction of homoduplex values and are shown in Figure 2G.

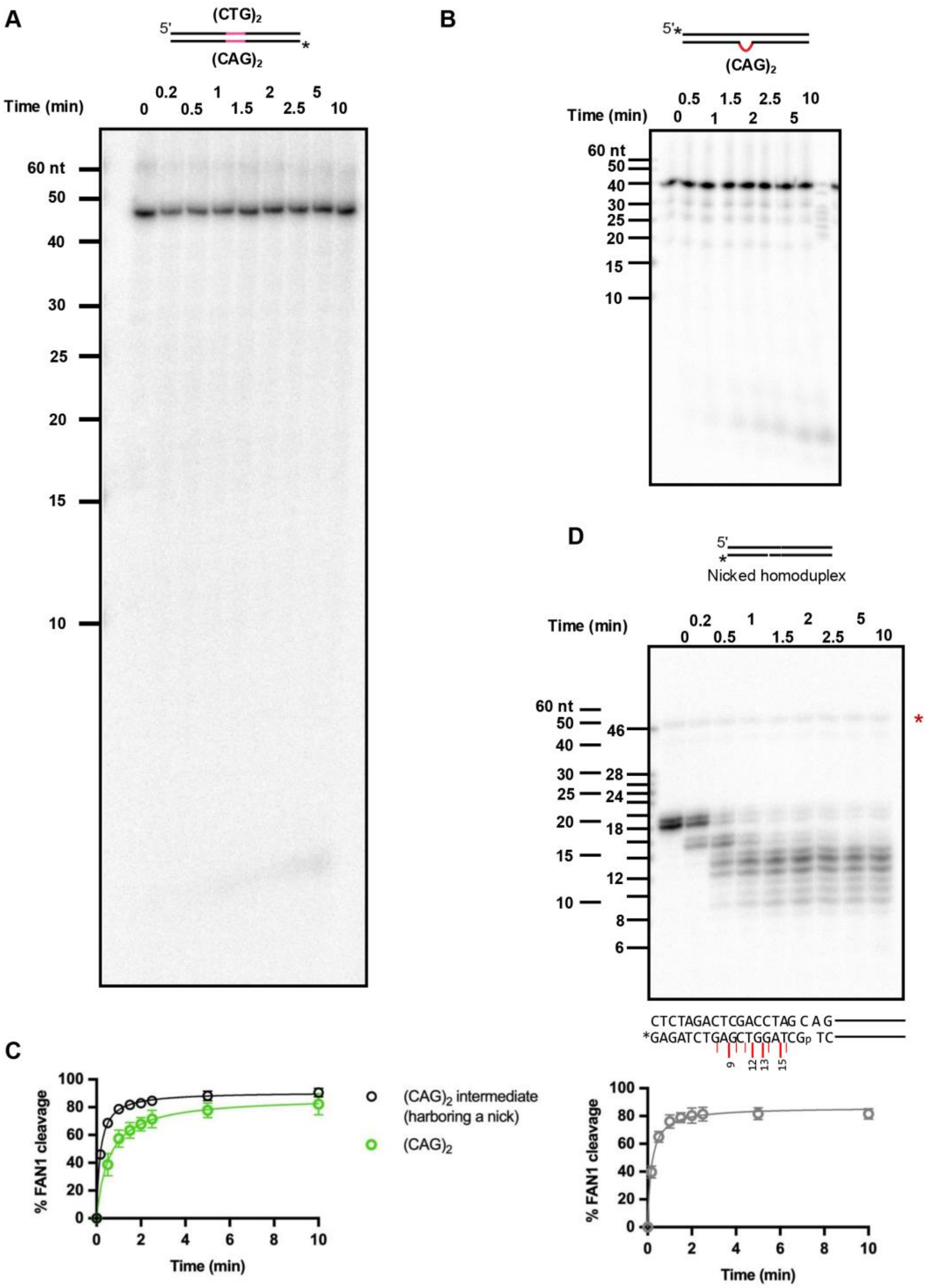

Supplementary Figure 4. (**A**) Five nM of 5'-radiolabeled homoduplex control or (**B**) DNA substrate harboring $(CAG)_2$ extrusion (that was 5'-radiolabeled on the complementary DNA strand), were incubated with 11 nM of FAN1 at 37ºC for indicated time in the presence of 70 mM KCl and 5 mM $MgCl_2$. Samples were collected and analyzed on 20% denaturing PAGE. The image is a representative of n=3 independent experiments. (**C**) Direct comparison of time dependent FAN1 activity on $(CAG)_2$ DNA substrate (data from Figure 2B) and $(CAG)_2$ substrate harboring a nick located 2 nucleotides 3' to the extrusion to mimic a DNA intermediate after the FAN1 endonuclease cleavage (data from Figure 3C). (**D**) A 3'- end radiolabeled homoduplex DNA harboring a single nick (control for a DNA substrate in Figure 3B); p indicates phosphorylation of the terminal nucleotide. Five nM of such DNA substrate was incubated with 11 nM FAN1, and samples were collected and analyzed as described in (A). Quantification based on 3 independent experiments with error bars representing SD. Red asterisk indicates not fully denatured DNA.

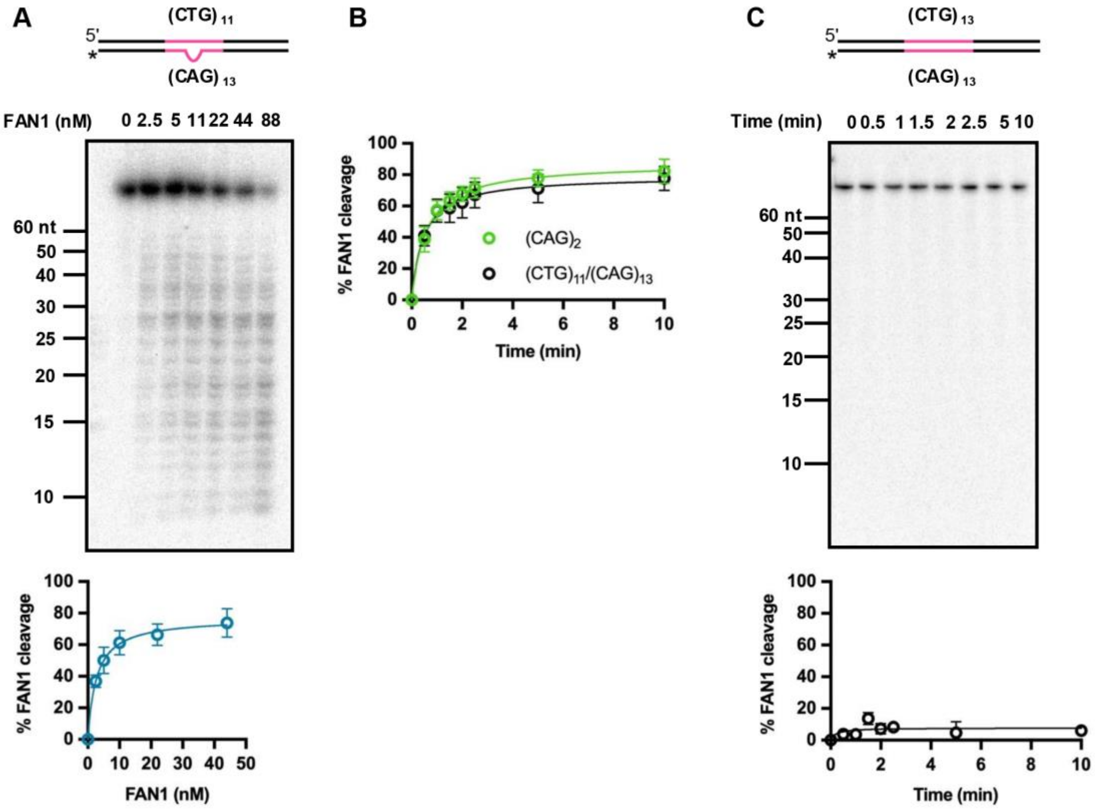

Supplementary Figure 5. (**A**) Five nM 3'-end radiolabeled $(CTG)_{11}/(CAG)_{13}$ DNA substrate was incubated with increasing concentrations of FAN1 at 37ºC in a buffer containing 70 mM KCl and 5 mM $MgCl_2$ for 10 min. Samples were collected and resolved on 20% denaturing PAGE. Quantification of percent of FAN1 cleavage is shown below. Graph represents mean values ± SD of n=5 independent experiments. (**B**) Direct comparison of time dependent FAN1 activity on $(CAG)_2$ DNA substrate (data from Figure 2B) and $(CTG)_{11}/(CAG)_{13}$ (data from Figure 4B). (**C**) Five nM 3'-end radiolabeled $(CTG)_{13}/(CAG)_{13}$ homoduplex DNA was incubated with 11 nM FAN1 at 37ºC in presence of 70 mM KCl and 5 mM $MgCl_2$. Samples were collected at indicated time points and analyzed as in (A). Quantification of percent of FAN1 cleavage is shown below. Graph represents mean values ± SD of n=3 independent experiments.
